# Prevalence and Associated Factors of Depression in Nepal: A Systematic Review and Meta-analysis

**DOI:** 10.31729/jnma.8960

**Published:** 2025-04-30

**Authors:** Pawan Sharma, Swarndeep Singh, Anima Parajuli, Omkar Dhungel, Pankaj Pathak

**Affiliations:** 1 Deparment of Psychiatry, Patan Academy of Health Sciences, Lagankhel, Lalitpur, Nepal; 2 Vardhman Mahavir Medical College and Safdarjung Hospital, New Delhi, India; 3 Snree Birendra Hospital, Chhauni, Kathmandu, Nepal; 4 Dadeldhura Hospital, Dadeldhura, Nepal

**Keywords:** *depression*, *prevalence*, *meta-analysis*, *Nepal*, *systematic review*

## Abstract

**Introduction:**

Depression is a common mental disorder associated with considerable degree of burden. This review aimed to examine the prevalence of depression and associated factors in the Nepalese general population.

**Methods:**

The databases Medline (PubMed), Embase, Scopus, Web of Science and NepJOL were searched for identification of peer-reviewed studies reporting the prevalence of depression among non-clinical populations in Nepal along with Google Scholar and citation search. Meta-analysis was conducted using random-effects model to calculate the pooled prevalence estimates. A qualitative synthesis of factors associated with depression was performed.

**Results:**

A total of 64 studies comprising a total of 57,553 participants were included. Overall, the pooled prevalence among adult population was 13.75% (95% CI:10.84% to 16.65%). For other population subgroups: 27.49% (95% CI: 17.99% to 32.21%) in children and adolescents, 50.07% (95% CI: 32.82% to 67.33%) in geriatric population, 19.96% (95% CI: 18.00% to 21.91%) in maternal population. There was high degree of heterogeneity (I2 = 99.32). Depression was associated with distinct individual attributes and behaviors, socio-economic circumstances and environmental factors.

**Conclusions:**

Approximately one-seventh of the adult population was found to have depression. There were notable variations in the prevalence and associated factors of depression across different population subgroups.

## INTRODUCTION

Depression is a leading cause of mental health related disability throughout the world.^1^ According to World Health Organization (WHO) report, the global prevalence of depression was estimated to be about 5%.^2^ The National Mental Health Survey of Nepal conducted in the year 2020, reported the prevalence of depression to be 0.6% among adolescents (13-17 years of age), and 1% in adults, with a lifetime prevalence of 2.9%.^3^

There is significant variation in the prevalence rates of depression in the available literature which could be due to studies conducted across different countries, study participants’ characteristics and other methodology or depression assessment related factors. In view of this, there was a need to conduct a systematic review of available studies assessing the prevalence of depression across different subgroups of Nepalese population. Thus, this review aimed to systematically analyze the available literature examining the prevalence of depression among non-clinical population subgroups in Nepal.

## METHODS

This systematic review was designed and carried out in accordance with the Preferred Reporting Items for Systematic Reviews and Meta-Analyses (PRISMA) guidelines.^4^ The review protocol was pre-registered in the International Prospective Register of Systematic Reviews (PROSPERO) (Registration Number: CRD42023433307).

A comprehensive electronic search for original articles published in English was conducted on PubMed, Embase, Scopus, Web of Science and NepJOL (Nepal Journal Online) since inception of database till December 2023. The keywords "depression”, "prevalence”, and "Nepal" were adapted. Additionally, Google Scholar search and manual screening of key local journals along with reference list of selected articles was also conducted. Two investigators (OD and PP) independently screened the titles and abstracts as per the eligibility criteria, and disagreements were resolved by majority consensus among other investigators. The full-texts of shortlisted studies were screened to confirm their eligibility. All the population studies, epidemiological studies, retrospective and prospective observational studies (cohort, cross sectional and case-control), experimental studies estimating prevalence of depression among non-clinical, community sample with data available on total number of sample size (denominator) and showing percentage or number of patients diagnosed with depression (numerator) in Nepal using either diagnostic interview or validated depression screening questionnaires from peer-reviewed publications in English language with full-text available were taken. The articles with qualitative outcomes and multiple articles from same projects/dataset with overlapping estimates of depression, review, editorial, erratum, conference abstracts, and letters, studies related to clinical and at-risk populations, surveys/studies that calculated prevalence based on the response to a single question were excluded. One Investigator (AP) performed the task of data extraction and the extracted data was independently cross examined by two other investigators (SS and PS) for accuracy. The prevalence of depression was taken as primary outcome variable, and other variables like type of study, sample size, region of study, participant characteristics, assessment tools and factors associated with depression were extracted.

The overall methodological quality of each study was evaluated and rated using the "Joanna Briggs Institute (JBI) Quality Assessment Tool for prevalence Studies".^5^ The included studies were classified into the following three categories based on their total JBI score percentages: low (at least 70%); moderate (50%-69%); and high (49% and below) risk of bias (ROB).^6^

Open Meta Analyst software was used for conducting meta-analyses of selected studies identified in the systematic review (http://www.cebm.brown.edu/openmeta). Random-effects meta-analysis along with appropriate sub-group and sensitivity analyses was conducted in keeping with the latest guidance provided for researchers conducting proportional meta-analyses.^7^

**Figure 1:**
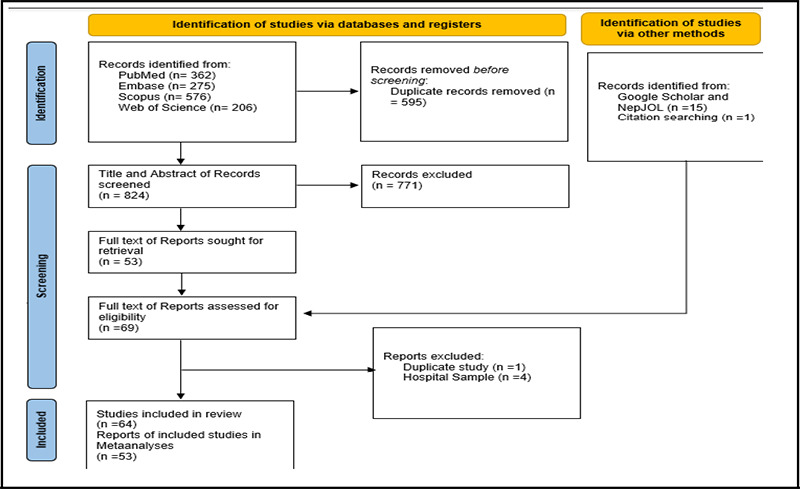
PRISMA 2020 flow diagram for selection of studies in the systematic review and meta-analysis

## RESULTS

A total of 64 studies representing a total of 57553 participants were included in the systematic review. Out of 64, 53 were also included in meta-analysis. Figure 1 depicted PRISMA flow diagram for study selection. A summary of studies included in this systematic review is given in Table 1^8-59^ and Table 2^60-70^ (Supplementary file 2). Overall, point prevalence of depression ranged from 1.00% to 82.60% across studies. Study sample sizes ranged from 62 to 10,714. Nine studies reported findings from adult populations, ten from geriatric population, thirteen from medical students, three from health care worker, eleven from child and adolescent population, seven from maternal population. As per JBI, 20 studies were categorized as high ROB, 18 as moderate ROB, and 26 as low ROB.

***Prevalence of depression in adults***

The prevalence of depression in adults across studies ranged between 1.00% and 34.11.^3,13^ The pooled prevalence of depression estimated using a random effects model was 13.75% (k= 10; 95% CI: 10.84% to 16.65%), accompanied with a high degree of statistical heterogeneity (I2 = 99.32%; p <0.001). Figure 2a depicts the forest plot for pooled prevalence of depression in the adult population. The removal of individual studies from the analysis conducted sequentially, did not substantially change the estimated pooled prevalence of depression among any of the above-described population sub-groups. The overall prevalence was 11.68% (95% CI: 8.89% to 14.46%) when the study by Kane et al. (2018) was excluded, and was 15.65% (95% CI: 10.47% to 20.82%) when the study by Dhimal et al. (2021) was excluded.^13,3^

The results of meta-regression analyses showed that the prevalence of depression among adults was not significantly affected by year of data collection, mean age of study participants, percentage of male in the study sample, JBI quality score, or response rate (all p-values >0.05).

In view of the high degree of heterogeneity and no significant factor identified in meta-regression analyses, we conducted exploratory sub-group metaanalysis across different population sub-groups by sub-dividing the studies based on the method used for detecting or diagnosing depression in them (i.e., screening/self-administered tool or confirmatory/clinician-administered tool). There was a significantly higher prevalence rate of depression reported in studies conducted among adult population when the researchers applied a screening or self-administered tool (21.53%; 95% CI: 15.12% to 27.94%) as compared those studies in which a confirmatory or clinician-administered tool (3.39%; 95% CI: 1.79 to 4.99%) was applied.

***Prevalence of depression in children and adolescents***

The prevalence of depression in children and adolescents across studies ranged between 0.60% and 56.51%.^3,24^ The pooled prevalence of depression estimated using a random effects model was 25.10% (k= 12; 95% CI: 17.99% to 32.21%), accompanied with a high degree of statistical heterogeneity (I2 = 99.50%; p <0.001). Figure 2b depicts the forest plot for pooled prevalence of depression in the pediatric population.

The removal of individual studies from the analysis conducted sequentially, did not substantially change the estimated pooled prevalence of depression among any of the above-described population sub-groups. The overall prevalence was 22.15% (95% CI: 15.61% to 28.69%) when the study by Karki et al. (2022) was excluded, and was 27.49% (95% CI: 18.62% to 36.36%) when the study by Dhimal et al. (2021) was excluded.^24,3^

The results of meta-regression analyses showed that the prevalence of depression among children and adolescent increased significantly with mean age of study participants (Coefficient= 0.091; p-value= 0.003).

***Prevalence of depression in geriatric population***

The prevalence of depression in geriatric population across studies ranged between 15.37% and 82.58%.^32,34^. The pooled prevalence of depression estimated using a random effects model was 50.07% (k= 10; 95% CI: 32.82% to 67.33%), accompanied with a high degree of statistical heterogeneity (I2 = 99.12%; p <0.001). Figure 2c depicts the forest plot for pooled prevalence of depression in the geriatric population.

The removal of individual studies from the analysis conducted sequentially, did not substantially change the estimated pooled prevalence of depression among any of the above-described population sub-groups. The overall prevalence was 46.45% (95% CI: 29.35% to 63.54%) when the study by Lamichhane et al. (2022) was excluded, and was 54.02% (95% CI: 40.15% to 67.88%) when the study by Thapa et al. (2020) was excluded.^32,34^

The results of meta-regression analyses showed that the prevalence of depression among elderly decreased significantly with percentage of males in study sample (Coefficient= -0.021; p-value= 0.005).

**Figure 2a:**
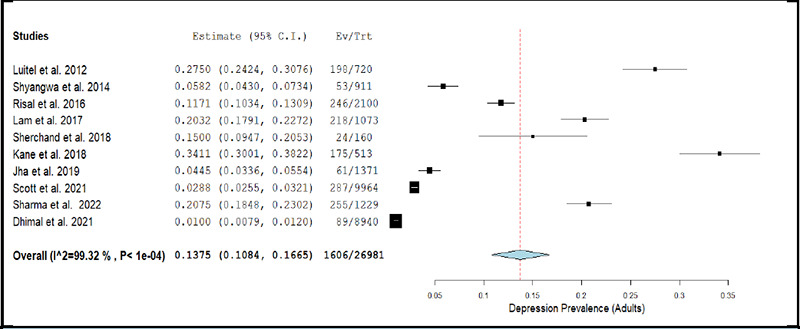
Forest Plot of studies reporting prevalence of depression in adults

**Figure 2b:**
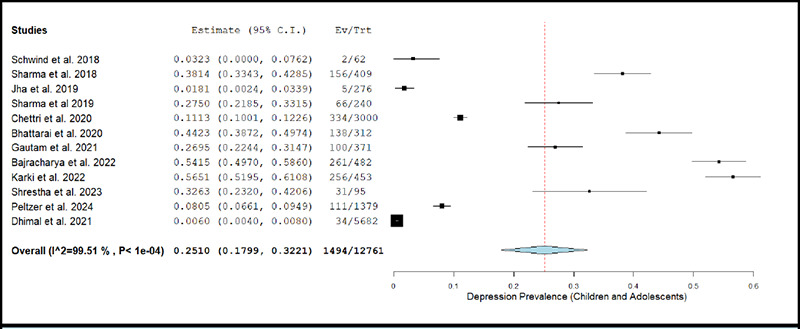
Forest Plot of studies reporting prevalence of depression in children and adults

**Figure 2c:**
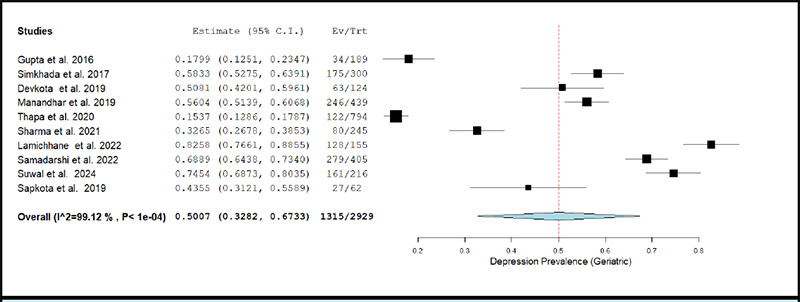
Forest Plot of studies reporting prevalence of depression in geriatric population

**Figure 2d:**
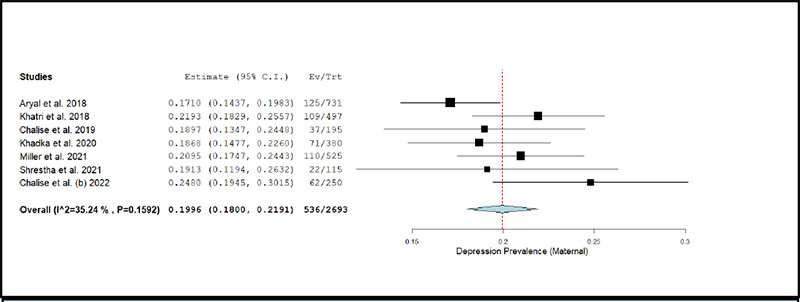
Forest Plot of studies reporting prevalence of depression in maternal population

**Figure 2e:**
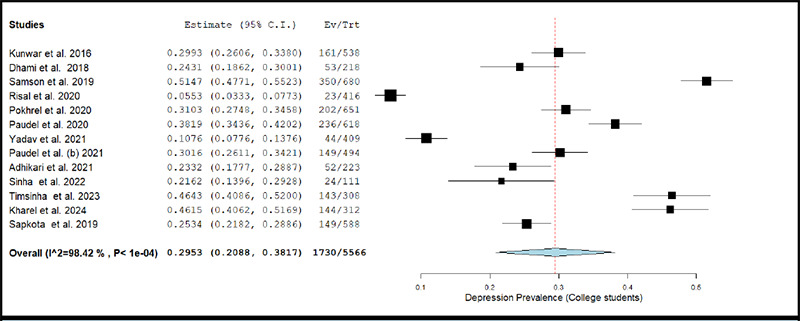
Forest Plot of studies reporting prevalence of depression in college student population

**Figure 2f:**
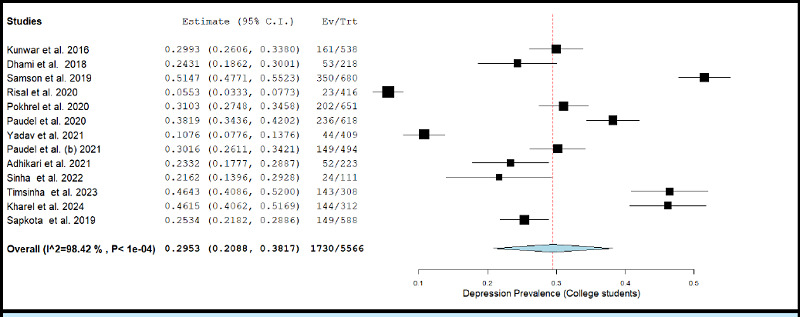
Forest Plot of studies reporting prevalence of depression in health care workers

***Prevlaence of depression in maternal population***

The prevalence of depression in maternal population across studies ranged between 17.10% and 24.80%.^37,43^. The pooled prevalence of depression estimated using a random effects model was 19.96% (k= 7; 95% CI: 18.00% to 21.91%), accompanied with a low degree of statistical heterogeneity (I2 = 35.24%; p= 0.159). Figure 2d depicts the forest plot for pooled prevalence of depression in the maternal population.

The removal of individual studies from the analysis conducted sequentially, did not substantially change the estimated pooled prevalence of depression among any of the above-described population sub-groups. The overall prevalence was 19.31% (95% CI: 17.65% to 20.97%) when the study by Chalise et al. (2022) was excluded, and was 20.82% (95% CI: 19.03% to 22.62%) when the study by Aryal et al. (2018) was excluded.^43,37^

The results of meta-regression analyses showed that the prevalence of depression among maternal population increased significantly with higher JBI score indicating lesser risk of bias (Coefficient= 0.012; p-value= 0.005).

***Prevalence of depression in college students***

The prevalence of depression in college students across studies ranged between 5.53% and 51.47%.^48,47^ The pooled prevalence of depression estimated using a random effects model was 29.53% (k= 13; 95% CI: 20.88% to 38.17%), accompanied with a high degree of statistical heterogeneity (I2 = 99.12%; p <0.001). Figure 2e depicts the forest plot for pooled prevalence of depression in the college student population.

The removal of individual studies from the analysis conducted sequentially, did not substantially change the estimated pooled prevalence of depression among any of the above-described population sub-groups. The overall prevalence was 27.67% (95% CI: 19.66% to 35.69%) when the study by Samson et al. (2019) was excluded, and was 31.57% (95% CI: 24.28% to 38.85%) when the study by Risal et al. (2020) was excluded.^47,48^

The results of meta-regression analyses showed that the prevalence of depression among college students was not significantly affected by either year of data collection, mean age, percentage of males in study sample, JBI quality score, or response rate (all p-values >0.05).

***Prevalence of depression in health care workers (HCWs)***

The prevalence of depression in HCWs across studies ranged between 28.96% and 41.31%.^53,58^ The pooled prevalence of depression estimated using a random effects model was 35.65% (k= 3; 95% CI: 28.58% to 42.71%), accompanied with a high degree of statistical heterogeneity (I2 = 83.15%; p =0.002). Figure 2f depicts the forest plot for pooled prevalence of depression in the college student population.

The removal of individual studies from the analysis conducted sequentially, did not substantially change the estimated pooled prevalence of depression among any of the above-described population sub-groups. The overall prevalence was 33.23% (95% CI: 24.88% to 41.57%) when the study by Pandey et al. (2020) was excluded, and was 38.63% (95% CI: 35.00% to 42.27%) when the study by Adhikari et al. (2021) was excluded.^53,58^

The results of meta-regression analyses showed that the prevalence of depression among HCWs was not significantly affected by the year of data collection response rate (p-value= 0.118). There was a significant association of depression prevalence with the mean age (Coefficient= -0.022), percentage of males in study sample (Coefficient= 0.008), JBI quality score (Coefficient= 0.097) of the study (all p-values <0.05).

## DISCUSSION

The current meta-analysis estimated the pooled prevalence of depression among adult population to be around 13.75%. This prevalence is comparable to the aggregate point prevalence of depression of 12.9% reported in a previous meta-analysis comprising of 90 community sample based studies from 30 different countries between the time period of 1994 and 2014.^71^ However, this pooled prevalence is less than the global point prevalence of 4.7% shown in another study after adjusting for methodological differences.^72^ In the current meta-analysis as well, the pooled prevalence estimate was 3.39% when confirmatory or clinician-administered tools were applied for assessment of depression in adult population, which is comparable to the adjusted global point prevalence of 4.7%.^72^ Another systematic review of reviews from South Asia noted that among 25 primary studies, pooled prevalence of depression was around 16.0%.^73^ However, the prevalence of depression among adult population as per the National Mental Health Survey of Nepal was 2.9%, which is considerably less than that obtained in the current meta-analysis.^3^ The high degree of variability observed in the prevalence of depression could be attributed to differences in the study methodology, tools used for diagnosis, technical vulnerabilities of existing diagnostic interviews and other cultural contexts.^74^ The associated risk factors for depression such as female gender, low educational level, being widowed and separated, low social support, financial hardships detected in our review are supported by the available research on social determinants of depression.^75,76^ Among sex workers, exposure to violence and belonging to sexual minorities such as transgender were noted as factors associated with depression in them. This is in line with the findings of a previous systematic review, that included population based studies assessing depression among youths of sexual minorities from predominantly Western countries.^77^

The pooled prevalence of depression among children and adolescents was estimated as 27.49%, which was much higher than the global pooled prevalence of 2.6% shown by a prior meta-analysis comprising of 41 studies conducted across 27 countries from across different regions of the world.^78^ Another review from Global Burden of Disease Study group showed the prevalence of depression to be around 6.2%.^79^ The reason for this high prevalence observed in our review could be due to the use of different assessment scales and use of scales with a lower threshold for diagnosis or a lower cutoff value (e.g., applying screening thresholds rather than more stringent diagnostic threshold used for making confirmatory diagnosis), and inclusion of cases of depression from mild to severe.^80^ Importantly, the prevalence rate ranged from 1.81% to 56.51%, showing a great degree of variation in prevalence across studies; similar to the data presented in a previous review of Indian studies assessing prevalence of depression among children and adolescents.^81^ The associated factors in this subgroup included dissatisfaction with academic performance and perceived academic stress, problematic internet use, sleep deprivation, substance use poor social support, low self-esteem and poor relationship with friends. These risk factors were in line with that reported from other studies conducted in the South Asian region.^82,83^

The pooled prevalence of depression among geriatric population was about 50.07%, which was higher than that reported in another meta-analysis comprising of 42 studies across different countries of the world. The pooled global prevalence among elderly in this prior meta-analysis was 31.74%, with sub-group metaanalysis reporting a significantly higher depression prevalence in studies from developing countries as compared to the developed countries (40.78% vs 17.05%).^84^ Another meta-analysis showed that the prevalence of depression among older adults was 28.4%; which varied with diagnostic tools, sample size and representativeness, and geographic region of studies included in the analysis.^85^ Our finding is slightly higher than the findings shown by similar studies conducted in South Asian (42.0%)^86^ and Indian context (34.4%).^87^ The associated factors of geriatric depression that have been shown in our study were similar to that reported in the global literature.^88^

The pooled prevalence of depression among maternal population subgroup was about 19.96%, which was comparable to the reported pooled prevalence of about 17% from a previous meta-analysis comprising of 58 studies assessing post-partum depression among healthy mothers with no prior history of depression. ^89^ Also, another meta-analysis reporting pooled prevalence of postpartum depression from 52 low and middle income countries reported similar prevalence estimate of 24.7%, which is again comparable to our estimated prevalence.^90^ The associated factors for maternal depression noted in our review are also in line with the published literature from South Asian countries, in which factors like unplanned pregnancy, intimate partner violence, male gender preference for newborn baby, and poor relationship with spouse or inlaws have been associated with maternal depression.^91^

The pooled prevalence of depression among college going students was about 29.53%, which is comparable to the finding of 30.6% from another systematic review of 24 studies,^92^ and 23.8% pooled prevalence estimate from a meta-analysis of 34 studies from China.^93^ Lastly, the pooled prevalence of depression among health care workers was 35.65%. Most of the studies assessing prevalence of depression among health care workers were done during or after the COVID-19 pandemic. Thus, they are likely to give biased results. A study done among 10,325 participants health workers found that about 30.2% of them were likely to meet the diagnostic criteria for clinical depression.^94^ A metaanalysis of 77 studies from East Mediterranean Region reported a pooled prevalence of 33.03% for depression among health care workers.^95^ These findings were somewhat similar to the results of our systematic review and meta-analysis.

To best of our knowledge, this is the first systematic review and meta-analysis to comprehensively assess published literature and estimate the depression prevalence across different population sub-groups in Nepal. The rigorous methodology adopted to conduct this review in a transparent and systematic manner in line with the PRISMA guidelines further adds to the reliability of our review findings. Furthermore, sensitivity analysis conducted as part of the metaanalyses revealed that none of the included studies substantially influenced or biased the overall results.

However, certain limitations that need to be kept in mind while interpreting our findings. There is a wide variation in methods employed for assessment of depression across different studies included in this review; hence, there is disparity in prevalence rates. The structured diagnostic interviews have been used in few studies only, and most of the studies have used tools or questionnaires for detecting depression which might lead to an overestimated depression prevalence. The reliability and validity of several tools employed in included studies have not yet been established in Nepalese setting, thus limiting the reliability of individual study findings as well as the present metaanalysis. The marked heterogeneity across studies limits the generalizability of results. Apart from this limited sample sizes in several of the included studies, cross-sectional nature of studies, diverse ethnic groups, data taken during various disasters like earthquake, civil war and COVID-19 pandemic are some other important shortcomings.

Various disasters like earthquake, civil war and COVID-19 pandemic are some of the issues that also need to be accounted for while interpreting the review findings.

## CONCLUSION

The overall pooled prevalence of depression among Nepalese population was 13.75% among adults, 27.49% among children and adolescents, 50.07% among geriatric population, 19.96% among maternal population, 29.53% among college going student and 35.65% among health care workers. There were notable variations in the prevalence and associated factors of depression across different population subgroups.

## Data Availability

Data associated with this study are available upon reasonable request from the corresponding author.
